# Updated Review of the Evidence Supporting the Medical and Legal Use of NeuroQuant^®^ and NeuroGage^®^ in Patients With Traumatic Brain Injury

**DOI:** 10.3389/fnhum.2022.715807

**Published:** 2022-04-08

**Authors:** David E. Ross, John Seabaugh, Jan M. Seabaugh, Justis Barcelona, Daniel Seabaugh, Katherine Wright, Lee Norwind, Zachary King, Travis J. Graham, Joseph Baker, Tanner Lewis

**Affiliations:** ^1^Virginia Institute of Neuropsychiatry, Midlothian, VA, United States; ^2^NeuroGage LLC, Midlothian, VA, United States; ^3^Department of Psychiatry, Virginia Commonwealth University, Richmond, VA, United States; ^4^Department of Radiology, St. Mary’s Hospital School of Medical Imaging, Richmond, VA, United States; ^5^Karp, Wigodsky, Norwind, Kudel & Gold, P.A., Rockville, MD, United States; ^6^Gentry, Locke, Rakes and Moore, LLP, Roanoke, VA, United States; ^7^Department of Neuroscience, Christopher Newport University, Newport News, VA, United States; ^8^Department of Undergraduate Studies, University of Virginia, Charlottesville, VA, United States

**Keywords:** concussion, postconcussive syndrome, mild TBI, neuroimaging, MRI, volumetry, NeuroQuant^®^, NeuroGage^®^

## Abstract

Over 40 years of research have shown that traumatic brain injury affects brain volume. However, technical and practical limitations made it difficult to detect brain volume abnormalities in patients suffering from chronic effects of mild or moderate traumatic brain injury. This situation improved in 2006 with the FDA clearance of NeuroQuant^®^, a commercially available, computer-automated software program for measuring MRI brain volume in human subjects. More recent strides were made with the introduction of NeuroGage^®^, commercially available software that is based on NeuroQuant^®^ and extends its utility in several ways. Studies using these and similar methods have found that most patients with chronic mild or moderate traumatic brain injury have brain volume abnormalities, and several of these studies found—surprisingly—more abnormal enlargement than atrophy. More generally, 102 peer-reviewed studies have supported the reliability and validity of NeuroQuant^®^ and NeuroGage^®^. Furthermore, this updated version of a previous review addresses whether NeuroQuant^®^ and NeuroGage^®^ meet the Daubert standard for admissibility in court. It concludes that NeuroQuant^®^ and NeuroGage^®^ meet the Daubert standard based on their reliability, validity, and objectivity. Due to the improvements in technology over the years, these brain volumetric techniques are practical and readily available for clinical or forensic use, and thus they are important tools for detecting signs of brain injury.

## Introduction

Brain imaging has become an increasingly important aspect of the clinical and forensic evaluation of patients with traumatic brain injury and other neuropsychiatric disorders. Accordingly, it is important to be sure that these methods meet rigorous scientific and legal standards (Simpson, 2012). Herein, we discuss how these issues apply to neuropsychiatric disorders commonly associated with legal proceedings, with a special focus on traumatic brain injury, magnetic resonance imaging (MRI) brain volume measurement, and the Daubert standard.

Note that this article is an updated version of one published previously (Ross et al., 2013b). Since then, there have been many significant advances in the area, justifying the need for this updated review; the newer information was added to this article. And in order to minimize redundancy, this article summarizes much of the background material noted in the previous publication; please see that publication for additional details.

Traumatic brain injury (TBI) serves as a useful clinical example for understanding these issues. Although the diagnosis of TBI, especially mild TBI, often is not based on objective signs of injury (Menon et al., 2010), objective signs are important for clinical and legal reasons. In contrast to patients with severe TBI, patients with mild or moderate TBI typically have few or no objective signs of injury. The structural brain scans of these patients usually are interpreted as being normal or unremarkable (Ross et al., 2013c,^2015^). Accordingly, the trier of fact (the jury or, in the absence of a jury, the judge) often will hear plaintiff experts and defense experts state opposite conclusions regarding whether the plaintiff/patient suffered a brain injury. If objective measures of brain injury were available, it would make it easier for the trier of fact to determine the truth.

Fortunately, over the past few years, several advancements in technology have allowed for increased ability to objectively measure the effects of brain injury, even in patients with mild TBI. Perhaps foremost among these are tools for measuring MRI brain volume. This article describes two closely related methods, NeuroQuant^®^ and NeuroGage^®^, and the evidence regarding their admissibility in court under the Daubert standard.

## History of Structural Brain Imaging

### Early History of Structural Brain Imaging

A tremendous number of articles have been published over the past 35 years in the area of MRI brain volume and neuropsychiatric disorders; this conclusion is supported by PubMed searches conducted on 02/14/2022 of the following search terms: (1) “MRI” and “brain volume”: 39,646 publications; (2) “MRI,” “brain volume” and “brain injury”: 2,621 publications; (3) “MRI,” “brain volume” and “traumatic brain injury”: 695 publications. This extensive research generally has found the following: (1) most brain disorders are characterized by abnormal brain volume; (2) most chronic or degenerative brain disorders are characterized by brain atrophy, although some are characterized by abnormal brain enlargement (see below regarding findings in patients with chronic mild or moderate TBI); and (3) greater degrees of brain volume abnormality often correlate with worse clinical symptoms or outcome.

These general findings also are true in TBI [for recent reviews, see Bigler (2021)]. Despite these research advances, MRI brain volumetry generally was not available in routine clinical settings until around 2007. What were the historical developments that led to the clinical availability of brain MRI volumetry?

In the early years of structural brain imaging (1970s to 1980s), research based on computed tomography (CT) scans and magnetic resonance imaging (MRI) scans mostly was based on visual inspection of the images. In the 1990s, researchers increasingly began to use computer-assisted methods to measure brain volume. However, brain volumetry was tedious and time-consuming and therefore limited to well-funded research settings.

### Development of Computer-Automated Brain Volumetric Tools

#### Intro to FreeSurfer

In the early 2000s, researchers developed more automated, computer-based methods for measuring brain volume. Several of these software programs currently are commonly used in research settings [for review, see Singh and Singh (2021)]. The current review will focus on FreeSurfer (Fischl, 2012) as a prominent example of these popular research tools, and as the cognate version of NeuroQuant^®^ (see below). We previously compared NeuroQuant^®^ and FreeSurfer (Ross et al., 2018a,^2021^) and will compare them briefly herein (see Table 1). In summary, FreeSurfer is free (as its name implies), flexible and popular in research settings. In contrast, NeuroQuant^®^ is commercially available, FDA-cleared, and has an integrated normal control database. Therefore, NeuroQuant^®^ is better suited for routine clinical application, although it is still quite useful for research. NeuroQuant^®^ has been found to have good to excellent reliability with FreeSurfer (Kovacevic et al., 2009; Ochs et al., 2015; Reid et al., 2017; Ross et al., 2018a; Chung et al., 2020; Wright et al., 2020); for a review of studies examining their reliability see section “Reliability of NeuroQuant^®^ and NeuroGage^®^” below.

**TABLE 1:** Comparison of NeuroQuant^®^ and FreeSurfer computer-automated MRI brain volumetry software programs.

Feature	NeuroQuant^®^	FreeSurfer	References
Free?	No	Yes	Fischl, 2012
Commercially available?	Yes	No	Birk, 2009; Fischl, 2012
FDA-cleared?	Yes	No	United States FDA [510(k) K061855]; https://www.accessdata.fda.gov/cdrh_docs/pdf6/K061855.pdf
Integrated normal control database?	Yes: >4,000 normal controls	No	
Initial learning curve	Easier	Harder	
Ease of use	Greater	Lesser	Heo et al., 2022
Flexibility	Less: e.g., regions with segmentation errors need to be excluded.	More: e.g., segmentation errors can be corrected manually.	Heo et al., 2022
Most common application	Mostly clinical, some research	Almost exclusively research	Ross et al., 2018a
Number of publications based on PubMed search done on 02/19/2022	102	2,316	
Total processing time	Shorter	Longer	Heo et al., 2022

*See Section “Development of Computer-Automated Brain Volumetric Tools” for discussion.*

#### Intro to NeuroQuant^®^. It Grew Out of FS

In response to these limitations of FreeSurfer, in the mid-2000s, scientists and clinicians at CorTechs Labs, Inc. (more recently, for marketing purposes called “CorTechs.ai”) developed NeuroQuant^®^, as essentially the portion of FreeSurfer that measured brain volume, customized for application in commercial settings (Birk, 2009; Fischl, 2011). Since then, NeuroQuant^®^ and FreeSurfer have evolved separately. In order to test the reliability and validity of NeuroQuant^®^, CorTechs Labs needed a sizable amount of normal control data collected using scientifically rigorous methods.

#### Alzheimer’s Disease Neuroimaging Initiative

Fortunately, other researchers were working to standardize MRI methods to optimize data collection and analysis and to encourage collaboration among researchers. A project in this area was the Alzheimer’s Disease Neuroimaging Initiative (ADNI), a consortium of researchers who collected MRI data and made them available for use by others. Although focusing on Alzheimer’s disease, this project included data from normal control subjects that could be used for comparisons with other neuropsychiatric patients. The ADNI normal controls were screened to exclude those with Alzheimer’s disease, significant impairment in cognitive functions or activities of daily living, Hachinski Ischemic Score of greater than 4 (i.e., at high risk of developing degenerative or vascular dementia), cortical strokes, a Geriatric Depression Scale score of greater than or equal to 6, substance abuse, and serious medical disease (including cancer or heart failure) (Petersen et al., 2010; Weiner et al., 2010). Although the normal controls were not screened specifically for traumatic brain injury (TBI), the screening criteria used would have excluded the large majority of people suffering from chronic effects of TBI.

A critical first step in the ADNI project was the development of a standardized image acquisition protocol that would be robust to intersite variation and would maximize the contrast between gray and white matter in the brain, an important consideration for the use of automated image analysis algorithms (Jack et al., 2008). The ADNI data allowed for the testing of the reliability and validity of NeuroQuant^®^ (Brewer, 2009).

#### NeuroQuant^®^ and Food and Drug Administration Clearance

In 2006, NeuroQuant^®^ was cleared for marketing by the United States Food and Drug Administration (FDA) [510(k) K061855] as a medical device “intended for automatic labeling, visualization and volumetric quantification of segmentable brain structures from a set of MR images”^1^. In other words, it was intended to automate the process of identifying MRI brain regions and measuring brain volume in human subjects^2^. NeuroQuant^®^ measures the volume of the whole brain and brain subregions and compares those volume to normal control subjects, adjusting for age, sex and intracranial volume. With the FDA’s ruling that it was essentially a “brain ruler” used to measure brain volume, its use is not restricted to any patient subgroup and it can be used in normal control subjects, patients with TBI, or other patients.

NeuroQuant^®^ was a major breakthrough for at least two reasons: (1) it reduced the time needed to identify brain regions from over 15 h to 10 min, greatly enhancing its practical utility, especially affordability; and (2) it is readily available and useful in typical clinical settings, unlike the software programs noted above that are used primarily in research university settings.

#### Intro to NeuroGage^®^

In 2012, NeuroGage^®^ was introduced by the Virginia Institute of Neuropsychiatry. NeuroGage^®^ is a software program that is built on NeuroQuant^®^ and extends its utility in several important ways, including evaluations of the following: (1) asymmetry (Ross et al., 2015, 2018b); (2) longitudinal change over time (Ross et al., 2012b,2013a, 2015, 2018b,^2021^); and (3) estimation of brain volume just before injury, based on the patient’s age, intracranial volume (measured from a later brain MRI; intracranial volume generally does not change during adulthood), and the known relationship between age, brain volume and intracranial volume during the normal adult lifespan (Ross et al., 2012b, 2013a, 2014, 2015, 2016, 2018b, 2021).

#### Evolution of NeuroQuant^®^ and NeuroGage^®^

In 2015, NeuroQuant^®^ 2.0 was released (followed by versions 2.1, 2.2, and 2.3), and in 2019, version 3.0 was released (followed by version 3.1 in May 2021) (for sample NeuroQuant^®^ 3.0 reports, see online supplementary file^3^).

Compared to earlier versions, NeuroQuant^®^ 3.0 has several improvements, including the following:

• More accurate segmentation algorithms, that is, algorithms for identifying brain subregions (for sample NeuroQuant^®^ 3.0 segmented brain images, see online supplementary file: see text footnote 3). Our clinical experience with hundreds of NeuroQuant^®^ 2.× and 3.0 analyses confirms that the segmentation is significantly more accurate; however, occasionally segmentation errors still occur, and therefore it remains important to inspect all results for possible segmentation errors (see below for further discussion of segmentation errors).
• Identification of more brain subregions (over 130).
• A larger normal control database: about 4000 normal controls ranging in age from 3 to 100 years. Additional information about the normal control database and its development and testing can be found at^4^.

NeuroGage^®^ 2.0 was similar to NeuroGage^®^ 1.0 insofar as it continued to provide analyses for asymmetry, longitudinal change and volume estimation (Ross et al., 2018b,^2021^). However, it was improved in several ways: (1) it was based on NeuroQuant^®^ 2.0 analyses instead of 1.0 analyses; (2) it included more brain regions than NeuroGage^®^ 1.0; (3) it included more normal controls (*N* = 80); and (4) and for determination of the normal relationship between brain volume vs. age across the adult lifespan, it used NeuroQuant^®^ normal control data (*N* = approximately 4000) instead of data from a previously published meta-analysis of normal control studies that in total had less normal controls (*N* = 2,211) and that used various volumetric software programs other than NeuroQuant^®^ (Hedman et al., 2012).

The latest version of NeuroGage^®^ software is 3.0, which is based on NeuroQuant^®^ 3.0 (Ross et al., 2021) (for sample NeuroGage^®^ reports, see online supplementary file: see text footnote 3). Compared to NeuroGage^®^ 2.0, version 3.0 was improved in the following ways:

• NeuroGage^®^ 3.0 includes a larger number of brain regions for asymmetry analyses (52 regions) and longitudinal analyses (e.g., t1–t2 analyses) (28 regions).
• For estimating brain volume, NeuroGage^®^ 3.0 includes a larger number of brain regions (10 regions), which was the number of brain regions that satisfied the requirement that all brain regions used for volume estimation have at least fair reliability [intraclass coefficient ≥ 0.5; (Koo and Li, 2016)]. Note that, as with previous versions of NeuroGage^®^, version 3.0 uses the brain volume estimation method to perform t0–t1 analyses, where brain volume is estimated at t0 (time of injury) and measured at t1 (time of the first NeuroQuanted MRI).
• NeuroGage^®^ 3.0 includes a biomarker test that accurately predicts group membership (normal controls vs. patients with chronic mild or moderate TBI) based on a single brain MRI scan. The test was developed using artificial intelligence methods, including neural networks (single layer) with a K-fold method for validation of results (to avoid overfitting) followed by a leave-one-out method for testing results [a more conservative method of avoiding overfitting; (Kocaguneli and Menzies, 2013)]. The final test had 100% sensitivity and 95% specificity. Additional details regarding the methods underlying the test are described in this supplementary file^5^.

## Use of NeuroQuant^®^ and NeuroGage^®^ in Legal Proceedings

### Court Cases in Which NeuroQuant^®^ and NeuroGage^®^ Were Admitted as Evidence

To date, NeuroQuant^®^ or NeuroGage^®^ have been admitted as evidence in 10 court cases, including one in federal court and one that produced a judge’s written Daubert ruling.

(1) *Burrell vs. Riverside Hospital*: Circuit Court of Newport News (VA), CL1101633F-15 (12/07/12). Per Avery T. Waterman, Jr., counsel for plaintiff, *via* email communication on 02/06/17, NeuroQuant^®^ brain volume data were admitted by consent agreement with defense counsel. The proceedings were not transcribed and the case was never appealed.
(2) *Frank J. Ferrante vs. City of Atlantic City, et al.*: Superior Court of New Jersey, Atlantic County Law Division (05/29/14). NeuroQuant^®^ findings were offered into evidence by plaintiff’s counsel. After hearing oral argument, the Hon. Michael Winkelstein issued an order denying defendants’ request to bar such testimony.
(3) *Federico, et al. vs. Mid-Atlantic Family Communities, LLC*: U.S.D.C. for the Eastern District of Virginia, Norfolk Division, Civil Docket No. 2:12cv80 (04/04/16). This case involved 2 family members who had neuropsychiatric sequelae from mold-related illness due to living in a damp, moldy home. According to the court transcript, Judge Jackson admitted NeuroQuant^®^ and NeuroGage^®^ brain volumetric evidence over objections of defense counsel.
(4) *An vs. Hekal*: Superior Court, Judicial District of Stamford/Norwalk (CT) (04/28/16). This case involved a woman who had mild traumatic brain injury. According to the court transcript, NeuroQuant^®^ and NeuroGage^®^ brain volumetric evidence and exhibits were admitted without objection by defense counsel.
(5) *Christopher Meskill vs. Kenri Ziko*: Connecticut Superior Court (05/02/17). NeuroQuant^®^ findings, including interval changes, were admitted as evidence by Judge Theodore Tyma. There was no challenge from the defense attorney, and there was no written court ruling regarding the NeuroQuant^®^.
(6) *Doupis vs. The City of New York et al.*: New York Supreme Court, County of New York (03/07/19). NeuroQuant^®^ findings were offered into evidence by plaintiff’s counsel. Defense counsel submitted a Motion in Limine to preclude plaintiff from offering into evidence the NeuroQuant^®^ findings or in the alternative, a Frye hearing. Justice Nervo denied the Motion and allowed the NeuroQuant^®^ findings into the case without a Frye hearing.
(7) *Donna O’Harren vs. Kaci Hedjar*: Williamsburg/James City County Circuit Court, Virginia (04/19/19). NeuroQuant^®^ and NeuroGage^®^ findings were admitted as evidence by Judge Michael McGinty. There was no challenge from the defense attorney, and there was no written court ruling regarding the NeuroQuant^®^ or NeuroGage^®^.
(8) *Shawn Donelson (father of R.D., minor) vs. Dustin Pointer*: Florida Circuit Court (07/02/21). This case involved a child with a head injury. Defense filed a Daubert Motion and argued that plaintiff could not satisfy its burden of establishing the reliability of the use of NeuroQuant^®^ because the normative database was unknown. Defendant also argued some lesser points, including that NeuroQuant^®^ did not provide a diagnosis. Following an evidentiary hearing Judge Charles Sniffen denied Defendant’s Daubert Motion (Judge Sniffen’s order is available here^6^).
(9) *Karen Bryant vs. Terry G Properties, LLC*: Common Pleas Court of Allen County, Ohio (08/12/21). This case involved a woman with a carbon monoxide-induced brain injury. Defense filed a motion to exclude NeuroQuant^®^ and NeuroGage^®^ results, arguing that they were neither scientific nor reliable. Following a Daubert hearing, Judge Jeffrey L. Reed denied defendant’s motion and allowed plaintiff’s expert to testify about the NeuroQuant^®^ and NeuroGage^®^ findings.
(10) *Abigail Chewning vs. Ashley Tye*: Powhatan Circuit Court (Powhatan County, VA) (09/17/21). This case involved a 23-year-old woman with moderate traumatic brain injury. NeuroQuant^®^ and NeuroGage^®^ findings were admitted as evidence by Judge Cella. There was no challenge from the defense attorney, and there was no written court ruling regarding the NeuroQuant^®^ and NeuroGage^®^ evidence.

To our knowledge, NeuroQuant^®^ and NeuroGage^®^ never have been excluded as evidence in court. More generally, we are not aware of any case in which the assessment of brain volume based on MRI has been excluded as evidence in court.

Given the increasing use of these brain volumetric tools (see below), it seems likely that NeuroQuant^®^ and NeuroGage^®^ will continue to be tested in court. Do they meet the standards required under federal law or under the many state law standards modeled on the federal approach? The following section will address this question with a focus on the Daubert standard.

### The Daubert Standard

Admission of expert testimony in federal court is governed by Rule 702 of the Federal Rules of Evidence. Numerous state courts have similar rules for expert witness testimony. Rule 702 provides:

A witness who is qualified as an expert by knowledge, skill, experience, training, or education may testify in the form of an opinion or otherwise if:

(a) the expert’s scientific, technical, or other specialized knowledge will help the trier of fact to understand the evidence or to determine a fact in issue;
(b) the testimony is based on sufficient facts or data;
(c) the testimony is a product of reliable principles and methods; and
(d) the expert has reliably applied the principles and methods to the facts of the case.

The judge, not the jury, makes the preliminary decision as to whether expert testimony is admissible under this rule. The task of the judge is not to determine whether the expert is correct or not, but to serve as a “gatekeeper” to guard against the admission of “junk science,” meaning opinions that are so unreliable as to be unworthy of consideration. The judge must determine whether the “expert is proposing to testify to (1) scientific knowledge that (2) will assist the trier of fact to understand or determine a fact in issue. This entails a preliminary assessment of whether the reasoning or methodology underlying the testimony is scientifically valid and of whether that reasoning or methodology properly can be applied to the facts in issue.” (Daubert and Rule 702).

Most litigation concerning the admissibility of expert testimony focuses on the standards under Rule 702(b), (c), and (d). The case which created these standards is Daubert vs. Merrill Dow Pharmaceuticals, Inc., 509 United States 579 (1993), and a typical motion to exclude an expert’s testimony in federal court is known as a “Daubert motion.”

In Daubert, the 9th Circuit Court of Appeals excluded an expert opinion that a particular drug caused birth defects. The testimony was based on scientific testing in animals and humans, as opposed to more familiar epidemiological studies. The scientific methodology was new and untried, and the 9*^th^* Circuit believed that it failed the legal test then used in federal court, known as the “general acceptance” test. The general acceptance test required that the scientific methodology be generally accepted by the scientific community in order for the scientific evidence to be admitted to court. The United States Supreme Court reversed the decision, and in doing so, did away with the general acceptance test in federal court and replaced it with a standard associated with Rule 702. Thus, under the Daubert standard, it is not necessary to show that an expert used a technique that is “generally accepted.”

Further, the focus of the analysis is on the way the expert arrived at the opinion, not whether it is correct. The latter question remains the province of the jury, which considers all admissible evidence to arrive at its verdict.

To be admissible, an opinion first must be based upon “scientific” knowledge, meaning that it was derived from a methodology “grounded in the methods and procedures of science.” Experts should be prepared through reports and in testimony to discuss not only their conclusions but also how the data support their conclusions considering, to the extent applicable and possible, the factors listed hereafter. But it is important not to conflate the use of percentages. In a forensic/civil legal setting, the burden of proof on any fact in dispute is the “preponderance of the evidence” which equates to greater than 50%. That standard in a civil case does not change even when scientific issues are involved. However, for a methodology to pass scientific approbation, it must meet appropriate scientific standards, for example, with a 95% confidence interval or rate of error that does not exceed 5%. Second, the opinion must assist the trier of fact in that it is relevant to an issue in dispute. Underlying both issues should be the recognition that the data reported by the NeuroQuant^®^ program are fully automated. In contrast, the data reported can and usually are interpreted by a clinician integrating the volumetric and other clinical data and applying customary techniques such as differential diagnosis.

The United States Supreme Court offered a list of suggested factors that a trial judge, acting as a gatekeeper, might examine in deciding whether to admit evidence. The list of factors is not exclusive, and it is not necessary for a court to consider them all in passing on a given opinion. This is especially true when an expert relies on scientific principles that are beyond question, such as immutable laws of nature. In practice, however, most motions to exclude expert testimony are based on a perceived failure to meet the “five factor test.”

Five key factors offered by the Supreme Court are as follows:

1. “[A] key question to be answered in determining whether a theory or technique is scientific knowledge that will assist the trier of fact will be whether it can be (and has been) tested.”
2. “Another pertinent consideration is whether the theory or technique has been subjected to peer review and publication.”
3. “Additionally, in the case of a particular scientific technique, the court ordinarily should consider the known or potential rate of error…”
4. “…and the existence and maintenance of standards controlling the technique’s operation.”
5. “Finally ‘general acceptance’ can yet have a bearing on the inquiry…. Widespread acceptance can be an important factor in ruling particular evidence admissible, and ‘a known technique which has been able to attract only minimal support within the community … may properly be viewed with skepticism.”

Other factors also may be relevant. Practitioners should consider as many potential factors as possible when assessing the viability of expert testimony. While no single factor is dispositive, most practitioners focus on the five Daubert factors. The following section will discuss the evidence regarding NeuroQuant^®^ and NeuroGage^®^ relevant to those five factors.

### Application of the Daubert Criteria to NeuroQuant^®^ and NeuroGage^®^

#### Daubert Factor 1: Testing the Theory

As discussed above, there have been thousands of scientific studies published over many years showing that MRI brain volume can be measured reliably and validly in normal people and people with neuropsychiatric disorders. The recent development of sophisticated volumetric software tools has made an accepted technique easier and faster; nevertheless, they basically still measure how big brain regions are and therefore remain essentially “brain rulers.”

#### Daubert Factors 2, 3 and 4: Reliability and Validity of NeuroQuant^®^ and NeuroGage^®^

##### Literature Review

**FIGURE 1:**
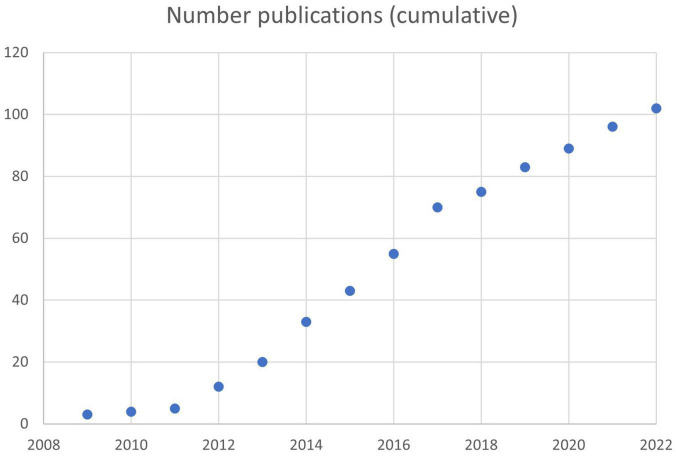
Rate of publication of peer-reviewed articles based on NeuroQuant^®^. A total of 102 articles had been published by 02/19/2022.

On 02/19/2022, searches were conducted using the term “NeuroQuant” on PubMed (including cross-referenced articles), Google and Google Scholar. 102 published peer-reviewed studies (described below) were identified that supported the reliability and validity of NeuroQuant^®^ for measuring brain volume in neuropsychiatric patients and normal control subjects (see Figure 1) (Brewer, 2009; Brewer et al., 2009; Kovacevic et al., 2009; McEvoy and Brewer, 2010, 2012; Heister et al., 2011; Engedal et al., 2012; Farid et al., 2012; Hampstead et al., 2012, 2016; Moen et al., 2012; Ross et al., 2012a,b, 2013a,b,c, 2014, 2015, 2016, 2018a,b, 2020, 2021; Bahar-Fuchs et al., 2013; Desikan et al., 2013; Hampstead and Brown, 2013; Ong et al., 2013; Rogne et al., 2013, 2016; Brezova et al., 2014; Elvemo et al., 2014, 2015; England et al., 2014; Evans et al., 2014; Hill et al., 2014; Kjelvik et al., 2014; Okamura et al., 2014; Shoemaker et al., 2014; Ting et al., 2014; Villemagne et al., 2014, 2017; Yaldizli et al., 2014; Yu et al., 2014; Azab et al., 2015; Bonner-Jackson et al., 2015; Braverman et al., 2015; Farlow et al., 2015; Fyock and Hampstead, 2015; Lam et al., 2015; Ochs et al., 2015; Saindane, 2015; Borba et al., 2016; Bredensen et al., 2016; Lyden et al., 2016; McMahon et al., 2016; Niemann et al., 2016; Pillai et al., 2016; Shankle et al., 2016; Wang et al., 2016; Graff-Radford et al., 2017; Kile et al., 2017; Leiva-Salinas et al., 2017; Min et al., 2017; Persson et al., 2017, 2018a,b; Reid et al., 2017; Relkin et al., 2017; Ritter et al., 2017; Seibert et al., 2017; Stelmokas et al., 2017; Tanpitukpongse et al., 2017; Ulstein and Bohmer, 2017; Vandenberghe et al., 2017; Eggins et al., 2018; Brinkmann et al., 2019; Cantó et al., 2019; Duma et al., 2019; Emrani et al., 2019; Ferrari et al., 2019; Kletenik et al., 2019; Pareto et al., 2019; Sudo et al., 2019; Chung et al., 2020; Louis et al., 2020; Vanier et al., 2020; Wright et al., 2020; Feng et al., 2020; Rothstein, 2020; Yim et al., 2020, 2021; Franceschi et al., 2021; Lee et al., 2021; Sabbagh et al., 2021; Soares et al., 2021; Bash et al., 2021; Bassal et al., 2021; Kim et al., 2021; Kwon et al., 2021; Morita-Sherman et al., 2021; Heo et al., 2022). Of these, 8 studies also used NeuroGage^®^ (Ross et al., 2012b, 2013a,c, 2014, 2015, 2018b, 2021).

##### Reliability of NeuroQuant^®^ and NeuroGage^®^

Because NeuroQuant^®^ is FDA-cleared, and because the FDA requires medical devices to be reliable, it can be assumed that NeuroQuant^®^ is reliable. Although this review process included submission of proprietary data that was reviewed by the FDA and might not have been made publicly available, there is ample data that was published, as discussed herein. Because the core process of NeuroQuant^®^ is fully computer-automated, its test-retest reliability on a single set of MRI images is 100% (confirmed by our own clinical experience with several cases).

With regard to different generations of NeuroQuant^®^ software versions, data within a generation (e.g., 2.0 and 2.3, or 3.0 and 3.1) have excellent reliability and can be mixed. However, data from different generations (e.g., 1.× and 2.×, or 2.× and 3.×) can have substantial differences due to improvements in the later software version(s), and therefore they should not be mixed. Stelmokas et al. confirmed this limitation, finding that two different generations of NeuroQuant^®^ software resulted in significantly different volumes; however, correlations between medial temporal lobe measures and neuropsychological variables generally did not differ between software versions (Stelmokas et al., 2017). More generally, other reports of reliability suggested that, if volumetry studies are conducted on different computing platforms or with different versions of software (e.g., FreeSurfer software), reliability should be tested across those platforms or software versions (Jovicich et al., 2009; Gronenschild et al., 2012).

There have been several studies of the intermethod reliability of NeuroQuant^®^. In a study of hippocampal volume, NeuroQuant^®^ was found to be reliable when compared with FreeSurfer (Kovacevic et al., 2009).

A more recent study of the reliability between NeuroQuant^®^ and FreeSurfer examined 21 brain regions and found high reliability for all regions except the pallidum and cerebellar white matter (Ochs et al., 2015). However, there often were large effect size differences between methods. Reid et al. published a similar study examining 30 brain regions and found similar results: high reliability for the large majority of brain regions, but frequent large effect size differences (Reid et al., 2017). These studies showed that NeuroQuant^®^ and FreeSurfer had high intermethod reliability, satisfying the Daubert criteria. High intermethod reliability means, for example, that if a group of patients–in comparison with a group of normal controls–were found to have brain atrophy using NeuroQuant^®^, then the same group of patients–in comparison with the same group of normal controls–likely would be found to have brain atrophy using FreeSurfer. The frequent occurrence of large effect size differences between the two methods indicated that they had low concurrent validity, as pointed out by Reid et al. This finding means that NeuroQuant^®^ and FreeSurfer results could not be mixed together because the results might not be valid. Therefore, generally it is preferable to use only one volumetric method for brain segmentation and volumetry, for example, for comparison of one or more patients to normal controls. Despite this limitation, is it possible to validly mix data based on the two methods? Yes, according to the study of Ross et al., which found that FreeSurfer brain volumetric data could be transformed into NeuroQuant^®^ values with high reliability and trivially small effect sizes using Bayesian regression, a machine learning technique (Ross et al., 2018a).

Two recent studies found that NeuroQuant^®^ and FreeSurfer showed fair to excellent reliability for all brain regions except the putamen (Yim et al., 2021) and pallidum (Yim et al., 2021; Heo et al., 2022).

Regarding other tests of the intermethod reliability of NeuroQuant^®^, it was found to be highly reliable with Structural Imaging Evaluation of Normalized Atrophy (SIENAX) (Wang et al., 2016). NeuroQuant^®^ and NeuroReader^®^ performed similarly with respect to using hippocampal volume to predict conversion of mild cognitive impairment to Alzheimer’s disease (Tanpitukpongse et al., 2017). Siemens software and NeuroQuant^®^ showed good-to-excellent inter-method reliability for most brain volumes except for the basal ganglia in patients with cognitive impairment (Chung et al., 2020). Comparisons of volume measurements between InBrain software and NeuroQuant^®^ showed good to excellent inter-method reliability for all brain regions except the pallidum (Lee et al., 2021).

Regarding tests of the reliability of NeuroQuant^®^ with manually or visually based methods, it has been found to be reliable with a computer-supported manual technique using NeuroMorphometric software (Brewer et al., 2009). NeuroQuant^®^ and the Scheltens scale (visual evaluation of medial temporal lobe atrophy) correlated highly and were similarly useful for distinguishing patients with Alzheimer’s disease from patients without dementia (Persson et al., 2018b). In a study of pediatric patients with TBI, Wright et al. found that Scheltens ratings of white matter hyperintensities (WMHs) had good to excellent agreement with WMH volumes for NeuroQuant^®^ (Wright et al., 2020); also NeuroQuant^®^ and FreeSurfer total white matter volumes correlated significantly and had fair agreement. Brinkmann et al. found that NeuroQuant-derived hippocampal volumes were more reproducible than hand-traced volumes (Brinkmann et al., 2019). In a study of hippocampal sclerosis in patients who underwent presurgical evaluation for temporal lobe epilepsy, NeuroQuant^®^ had specificity similar to that of the method of visual inspection by experts in radiological signs of hippocampal sclerosis; however, NeuroQuant^®^ had lower sensitivity, due in part to the fact that the radiology experts used T1- and T2-weighted images, whereas NeuroQuant^®^ is limited to using only T1-weighted images (Louis et al., 2020).

The test-retest reliability of NeuroQuant^®^ for 20 regions was examined in a group of 20 normal controls subjects (Ross et al., 2012b). Analyses showed excellent reliability for all regions except the ventral diencephalon, which had poor reliability.

NeuroQuant^®^ also was found to have excellent test-retest reliability in a sample of patients most of whom had epilepsy (Brinkmann et al., 2019).

NeuroQuant^®^ 2.0 compared to 2.3 software versions showed excellent reliability for all brain regions, indicating that the volume data were interchangeable between those software versions (Ross et al., 2021).

Since NeuroGage^®^ is based on NeuroQuant^®^, theoretically it shares NeuroQuant’s generally excellent reliability with respect to segmentation and volume measurement. We tested this idea directly in our normal control participants for multiple cortical and subcortical regions and found generally excellent test-retest reliability for NeuroGage^®^ 1.0 (*N* = 20 normals) (Ross et al., 2012b) and NeuroGage^®^ 2.0 (*N* = 80 normals) (Ross et al., 2018b). Brain regions with poor test-retest reliability, which occurred rarely, were not included in any version of NeuroGage^®^.

In addition to testing the reliability of NeuroGage^®^ with respect to volume measurement, it also was tested with respect to volume estimation. NeuroGage’s method for estimating brain volume is based on intracranial volume (which is stable throughout the adult lifespan) and brain volume-vs.-age growth curves throughout the adult lifespan (Ross et al., 2014, 2016, 2021). In the NeuroGage^®^ normal control group, estimates of brain volume were found to be highly reliable with measurements of brain volume for relatively large brain regions for NeuroGage^®^ 1.0 (Ross et al., 2014) and NeuroGage^®^ 2.0 (Ross et al., 2021).

In summary, the reliability of NeuroQuant^®^ and NeuroGage^®^ have been tested in multiple ways and found to be consistently good to excellent.

##### Validity of NeuroQuant^®^ and NeuroGage^®^

What is the validity of NeuroQuant^®^ and NeuroGage^®^? A basic aspect of validity is the ability of NeuroQuant^®^ to accurately identify, i.e., “segment,” brain subregions. [For examples of segmentation errors, see Ross et al. (2013c) p. 35, Figure 1].

For NeuroQuant^®^ 1.×, segmentation error rates ranged from 1.1% in patients with mild cognitive impairment (Heister et al., 2011) to 10.2% in patients with chronic mild or moderate traumatic brain injury (Ross et al., 2013c).

For NQ 2.× software, segmentation error rates ranged from 0.0% in normal controls and 1.5% in 55 patients with chronic mild or moderate TBI (Ross et al., 2021) to 18% in a sample of patients most of whom had epilepsy and temporal lobe asymmetry using NQ 2.0.1 hippocampal volumes (Brinkmann et al., 2019); the latter study included some patients with gross anatomical deformities, which are known to frequently cause segmentation errors.

In summary, segmentation error rates with NeuroQuant^®^ generally were low, especially with later versions of NeuroQuant^®^; but it appeared to be higher in patients than in normal controls, especially patients with gross anatomical deficits. Therefore, it remains important to inspect all segmented DICOMs for segmentation errors, and to exclude volume analyses based on brain regions that are not identified accurately.

Have NeuroQuant^®^ and NeuroGage^®^ been found to be useful in understanding brain disorders? The vast majority of the NeuroQuant^®^ published studies, referenced above in section “Literature Review: Segue to Peer-Reviewed Studies on Reliability and Validity. Total # Pubs: 102,” supported the conclusion that NeuroQuant^®^ was valid for assessing a variety of brain disorders, including Alzheimer’s disease, mild cognitive impairment, non-Alzheimer’s dementia, vascular dementia, traumatic brain injury, mold-related illness, temporal lobe epilepsy, multiple sclerosis, chronic pain, posttraumatic stress disorder, and others.

More specifically, studies of patients with traumatic brain injury using NeuroQuant^®^ or NeuroGage^®^ found that the patients had abnormal cross-sectional volumes (Ross et al., 2012a, 2013c, 2014, 2015, 2018b, 2019, 2020; Brezova et al., 2014), asymmetries (Ross et al., 2015, 2018b), and longitudinal brain volume changes (Ross et al., 2012b, 2013a, 2014, 2015, 2016, 2021). NeuroQuant^®^ and NeuroGage^®^ were found to be much more sensitive for detecting volume abnormalities than was the radiologists’ traditional technique of simple visual inspection (Ross et al., 2013c,^2015^).

More specifically, outpatients suffering from chronic effects of mild or moderate TBI were found to have some atrophy but more regions of abnormal enlargement (Ross et al., 2014, 2016, 2018b,^2020^) (for an example of a patient who had a pattern of cross-sectional brain volumes typical of chronic mild TBI, see the sample NeuroQuant^®^ and NeuroGage^®^ t1 and t2 reports in this online supplementary file: see text footnote 3). Multiple brain regions continued to enlarge over time, suggesting that the cross-sectional abnormal enlargement was not due simply to pre-injury enlargement (Ross et al., 2021) (for an example of a patient who had a pattern of longitudinal volume changes typical of chronic mild TBI, see the sample NeuroGage^®^ t1–t2 report in this online supplementary file: see text footnote 3). Other studies of patients with mild TBI also have found abnormal enlargement (Wang et al., 2015; Govindarajan et al., 2016). These findings were surprising because most studies of brain volume in patients with TBI have found extensive brain atrophy but not enlargement (Bigler, 2005, 2011), but most of those studies were based on patients with severe TBI. Taken together, these studies support the idea that chronic mild TBI has a pathophysiology that is at least somewhat different from that of severe TBI; in other words, it is not simply a milder version of severe TBI.

Several studies of TBI patients found significant correlations between NeuroQuant^®^ or NeuroGage^®^ volume measures and clinical symptoms or outcome. Greater rates of atrophy correlated with worse clinical outcome, including decreased ability to return to work or normal social relationships (Ross et al., 2012b; Brezova et al., 2014). Brain volume abnormalities correlated with acute measures of injury (including greater duration of posttraumatic amnesia and lower GCS score) and presence of diffuse axonal injury (Brezova et al., 2014). Vanier et al. (Vanier et al., 2020) divided a sample of mild TBI patients into those with and without MRI brain abnormalities, including hippocampal atrophy or asymmetry based on NeuroQuant^®^ analyses; patients with MRI abnormalities had slower recovery from balance and cognitive deficits. In a study of pediatric patients with TBI, Wright et al. found that increased volumes of white matter hyperintensities measured by NeuroQuant^®^ correlated with decreased cognitive processing speed (Wright et al., 2020). And longitudinal enlargement of the posterior cingulate gyrus was associated with the diagnosis of neuropathic headaches in patients with chronic mild or moderate TBI (Ross et al., 2021); this finding partially replicated an earlier finding by another group (Niu et al., 2020).

#### Daubert Factor 4: Maintenance of Standards and Controls

Additional information regarding maintenance of standards and controls can be provided by discussing our experience at the Virginia Institute of Neuropsychiatry and NeuroGage LLC. Since 2010, we have performed hundreds of NeuroQuant^®^ and NeuroGage^®^ analyses on our own patients and patients referred from outside physicians. Previously we described these procedures in detail (Ross et al., 2013b); therefore, this section will summarize and update the previous description. Examples of the application of quality control measures are shown in the sample NeuroQuant^®^ and NeuroGage^®^ reports at this supplementary online location: see text footnote 3.

Our quality control procedures include the following:

Before NeuroQuant^®^ processing:

• Prior to the collection of MRI data, we talk with personnel at the radiology center that will perform the MRI, and we explain the need to collect the data according to the NeuroQuant^®^/ADNI protocol. As part of that process, we refer the radiology personnel to the NeuroQuant^®^ website which explicitly states the scanner-specific parameters needed for entry into the MRI scanner^7^.
• Each MRI is interpreted by the attending radiologist in the traditional manner, that is, by visual inspection. The radiologist’s interpretation, along with the associated NeuroQuantable grayscale images, are reviewed by one of the co-authors (D.E.R.), who is board-certified in neuropsychiatry and brain injury medicine. Particular attention is paid to structural brain abnormalities or other factors, e.g., motion artifact that could affect brain volumetry. Based on that analysis, a decision is made regarding whether to submit the grayscale imaging for NeuroQuant^®^ processing.

During NeuroQuant^®^ processing:

• The NeuroQuant^®^ software automatically checks several parameters in order to ensure that the MRI data were collected accurately. If not, the submission will be rejected by the computer and the analysis will not be performed. These parameter checks include the following:
• The MRI scan must have been collected as T1, sagittal, non-contrast, 3D.
• The Measurement Index (MI) must be <=5 for FDA compliance and clinical use. The MI is the co-efficient of “goodness of fit” to the internal atlas.

After NeuroQuant^®^ processing:

• Inspect the segmented DICOM images (colored brain images) for segmentation errors (for an example of a set of NeuroQuant^®^ 3.0 segmented DICOM images, see online supplementary file: see text footnote 3). If any region is identified inaccurately, the volumetric data associated with that region are not used in subsequent analyses.
• NeuroQuant^®^ 3.0 introduced a new tool, the Compatibility Assessment report, to accompany all volumetric analyses (for examples, see online supplemental sample file: see text footnote 3). This report analyzes several imaging parameters, flags parameters that were not set within 10% tolerance, and reports a global rating of compliance with recommended settings. Based on our experience with dozens of NeuroQuant^®^ 3.0 analyses, and email communication on 01/06/21 with Micki Maes, CorTechs Labs Clinical Operations Manager, we recommended the following guidelines for applying the results of the Compatibility Assessment report: (1) it is a useful tool that can help point out problems with scanner parameters but is not always diagnostic of whether the results are accurate; (2) visual inspection of the segmented DICOMs generally is a better way to determine whether the results are accurate; and (3) reviewing the Compatibility Assessment report and visually inspecting DICOMs is a better approach than either one alone.
• The numerical and statistical results of the analyses are inspected and compared with the segmented brain images to ensure validity. For example, in general, regions identified as abnormally large by visual inspection of the images should not be identified as abnormally small by the numerical analyses.
• The possibility of false positive findings should be considered in the interpretation of the results. For a given brain region, since the cutoff for abnormal volume is set at the 5*^th^* normative percentile for diminution and 95*^th^* normative percentile for enlargement, it would be expected that a typical healthy person would have 5% chance of having abnormally small volume and 5% chance of having abnormally large volume. If many brain regions are tested, 5% of those regions would be expected, for example, to have abnormally large volume. As a more specific example, the Triage Brain Atrophy report analyzes 135 regions; 5% of 135 = 6.8; and therefore approximately 7 regions would be expected to be abnormally large for a typical healthy person. If a given patient had more than 7 abnormally large regions, that finding would provide more support for the idea of an underlying brain disorder than a finding of less than 7 abnormally large regions.
• The pattern of volume findings should be subjected to the method of differential diagnosis. Since there is extensive literature on brain volume findings in patients with brain disorders, as discussed above, the interpreting physician should consider whether the pattern matches one disorder better than others.
• Following the general rule of radiological interpretation, the patient’s history should be used to help interpret volume findings. For a given patient suspected of having a given brain disorder but who does not have many volume abnormalities, a pattern of findings highly consistent with the pattern known to characterize that brain disorder still might provide support for that disorder.

In summary, although the core process of NeuroQuant^®^ volumetry is completely computer-automated, there is room for error both prior to the submission of brain MRI data for NeuroQuant^®^ analysis, during the NeuroQuant^®^ analysis process, and after the NeuroQuant^®^ automated analysis has been completed. Therefore, it is important that radiology centers and clinicians use quality control measures similar to those described above, in order to ensure the reliability and validity of the results.

#### Daubert Factor 5: Acceptance in the General Scientific Community

Regarding NeuroQuant’s acceptance in the general community, the most important indicator is its clearance by the United States FDA in 2006. Since then, its use has grown steadily: NeuroQuant^®^ analyses have been performed in “over 1,000 clinical sites in 35 countries processing over 1,000,000 cases to date” (^8^ accessed on 04/18/21).

Since its initial development in 2011, NeuroGage^®^ analyses have been conducted on approximately 500 patients and normal control participants from the United States and other countries.

As reviewed above, there have been 102 peer-reviewed publications using NeuroQuant^®^ (Figure 1) including 8 using NeuroGage^®^. To our knowledge, there have been no publications raising concerns about the reliability or validity of NeuroQuant^®^ or NeuroGage^®^.

#### Conclusions Regarding NeuroQuant^®^, NeuroGage^®^ and the Daubert^®^ Standard

Regarding the 5 Daubert factors discussed above, the following conclusions can be made. NeuroQuant^®^ and NeuroGage^®^ are based on the tested and well-accepted theories that brain disorders often cause brain volume abnormalities, and the extent of the abnormalities can be measured. These methods improve upon previous techniques because they are faster and more practical. Their reliability and validity have been supported by numerous peer-reviewed publications. The error rate has been tested and found to be acceptably low. Their use requires maintenance of certain standards and controls, but such standards have been found to be quite achievable. Their general acceptance in the scientific community has been evidenced by NeuroQuant’s FDA clearance and growing use of NeuroQuant^®^ and NeuroGage^®^. These data provide an adequate basis for the admissibility of NeuroQuant^®^ and NeuroGage^®^ evidence under federal law as well as under the many state law standards modeled on the federal approach.

## General Conclusion

In summary, NeuroQuant^®^ and NeuroGage^®^ have been proven to be valid, reliable, and practical means of measuring MRI brain volume. Accordingly, they are important tools for objectively assessing the effects of brain injury or disease on patients in clinical or medicolegal settings.

To understand the importance of this conclusion with regard to clinical or forensic application, consider a typical patient suffering from chronic effects of mild or moderate TBI. Such a patient’s diagnosis of TBI (Menon et al., 2010) and related symptoms is based mostly if not completely on subjective symptoms, e.g., “I have trouble remembering things” or “I feel fatigued every day.” The patient’s brain MRI images likely appear normal based on the radiologist’s traditional method of simple visual inspection (Ross et al., 2013c,^2015^), and the results of other objective testing (e.g., blood work, EEG) also are likely to be normal. The combination of subjective symptoms and normal MRI and other test results not infrequently leads to other people, including health care practitioners, doubting the veracity of the patient’s report. In cases like this, reliable and valid methods such as NeuroQuant^®^ and NeuroGage^®^ are likely to show multiple brain volume abnormalities (Ross et al., 2013c,^2015^), which can be critically important for helping other people understand the truth about what happened to the patient.

## Author Contributions

DR contributed to primary author. JS, DS, JBar, and TL contributed to imaging data collection and analysis. JMS contributed to data collection and review of legal section. JBak and KW contributed to imaging data collection and analysis and preparation of sample reports. LN, ZK, and TG contributed to authored parts of legal section. All authors contributed to the article and approved the submitted version.

## Conflict of Interest

DR is the sole owner and CEO of NeuroGage LLC, which produces NeuroGage^®^ software. In 2017, DR was a paid consultant for CorTechs Labs, Inc., which produces NeuroQuant^®^ software. DR serves as a forensic consultant for plaintiff and defense cases. TG is employed by Gentry, Locke, Rakes and Moore, LLP. The remaining authors declare that the research was conducted in the absence of any commercial or financial relationships that could be construed as a potential conflict of interest.

## Publisher’s Note

All claims expressed in this article are solely those of the authors and do not necessarily represent those of their affiliated organizations, or those of the publisher, the editors and the reviewers. Any product that may be evaluated in this article, or claim that may be made by its manufacturer, is not guaranteed or endorsed by the publisher.

## Footnotes

1
https://www.accessdata.fda.gov/cdrh_docs/pdf6/K061855.pdf

2
https://www.cortechs.ai/products/neuroquant

3
https://www.dropbox.com/sh/cqr0rqmx1o1hmpg/AAAbfFGEeHNNlI5dBoWidyJ8a?dl=0

4
https://www.cortechslabs.com/wp-content/uploads/2018/10/Normative-Database-White-Paper.pdf

5
https://www.dropbox.com/s/xqygpbkk3aaws8l/Methods%20of%20BM%20test_041721.docx?dl=0

6
https://www.dropbox.com/sh/3n9e1g15q0bz21a/AAD32T4NhgFSpIgoKfcUz_jHa?dl=0

7
https://www.cortechslabs.com/resources/scanner_setup

8
https://www.cortechs.ai/cortechs-labs-announces-company-rebrand-to-cortechs-ai-at-rsna-2020
